# Hydraulic hydrogel actuators and robots optically and sonically camouflaged in water

**DOI:** 10.1038/ncomms14230

**Published:** 2017-02-01

**Authors:** Hyunwoo Yuk, Shaoting Lin, Chu Ma, Mahdi Takaffoli, Nicolas X. Fang, Xuanhe Zhao

**Affiliations:** 1 Department of Mechanical Engineering, Massachusetts Institute of Technology, Cambridge, Massachusetts 02139, USA; 2 Department of Civil and Environmental Engineering, Massachusetts Institute of Technology, Cambridge, Massachusetts 02139, USA

## Abstract

Sea animals such as leptocephali develop tissues and organs composed of active transparent hydrogels to achieve agile motions and natural camouflage in water. Hydrogel-based actuators that can imitate the capabilities of leptocephali will enable new applications in diverse fields. However, existing hydrogel actuators, mostly osmotic-driven, are intrinsically low-speed and/or low-force; and their camouflage capabilities have not been explored. Here we show that hydraulic actuations of hydrogels with designed structures and properties can give soft actuators and robots that are high-speed, high-force, and optically and sonically camouflaged in water. The hydrogel actuators and robots can maintain their robustness and functionality over multiple cycles of actuations, owing to the anti-fatigue property of the hydrogel under moderate stresses. We further demonstrate that the agile and transparent hydrogel actuators and robots perform extraordinary functions including swimming, kicking rubber-balls and even catching a live fish in water.

## Abstract (web summary)

Hydrogel actuators have been widely developed to be osmotic-driven but many are in fact only capable of producing low forces. Here, the authors developed high speed and high force hydrogel actuators capable of camouflage optically and sonically with low fatigue over multiple cycles.

Transparent sea animals such as leptocephali (transparent larvae of eels) possess amazing capabilities of agile motions and optical and sonic camouflage in water, as their tissues and organs are mainly composed of active transparent hydrogels^1^,^2^. Owing to hydrogels' soft, wet and biocompatible nature, hydrogel-based actuators and robots that can imitate leptocephali's capabilities will enable transformative applications in areas as diverse as biomedicine^3^, soft robotics^4^,^5^, tunable optics^6^, and soft electronics and machines^7^,^8^,^9^,^10^. Actuations of existing responsive hydrogels mostly rely on their swelling/de-swelling driven by osmotic-pressure changes in response to external stimuli such as temperature^11^, pH (ref. 12), electric field^13^, light^14^ and ionic strength^15^. However, achieving both high-speed and high-force actuations in osmotic hydrogel actuators has been a great challenge due to the intrinsic coupling between the responsive time and the actuation force of hydrogels. As a result, osmotic-driven hydrogel actuators are limited by low actuation speed (for example, responsive time of minutes to hours) and/or low actuation force (for example, millinewtons)^11^,^12^,^13^,^14^,^15^,^16^. In addition, while cephalopod-inspired colouration has been intensively explored as a camouflage strategy for soft actuators and robots^5^,^10^,^17^,^18^, the active colouration requires prior information of the background colours in order to match with them. On the other hand, the optical and/or sonic transparency used by leptocephali represents a passive camouflage strategy naturally applicable in all backgrounds, potentially superior to active colouration. However, the leptocephali-inspired camouflage has not been well studied or exploited for soft actuators and robots yet^1^.

Here we report that hydraulic actuations of hydrogels with designed structures and properties can give soft actuators and robots that can achieve much higher actuation force (that is, over 1 N) and speed (that is, responsive time less than 1 s) than existing osmotic hydrogel actuators, and are capable of optical and sonic camouflage in water owing to their high transparencies. These bioinspired hydrogel-based systems are first steps towards mimicking the materials and functions, although not the exact anatomies, of leptocephali and other agile transparent sea animals^1^,^2^. While these animals typically use muscles to achieve active motions^1^,^2^, we adopt hydraulic actuation of hydrogels to give actuation forces and/or speeds much higher than existing osmotic hydrogel actuators and muscle-powered bioactuators^19^,^20^,^21^. We also demonstrate that the agile and transparent hydrogel actuators and robots perform extraordinary functions including fish-like swimming, kicking rubber-balls and catching a live fish in water. The current study not only addresses the long-lasting challenge of high-speed, high-force hydrogel actuators and robots but also introduces a new strategy to achieve passive camouflage in water by exploiting the optical and sonic transparency of hydrogels in aqueous environment.

## Results

### Osmotic and hydraulic actuation of hydrogels

**Figure 1:**
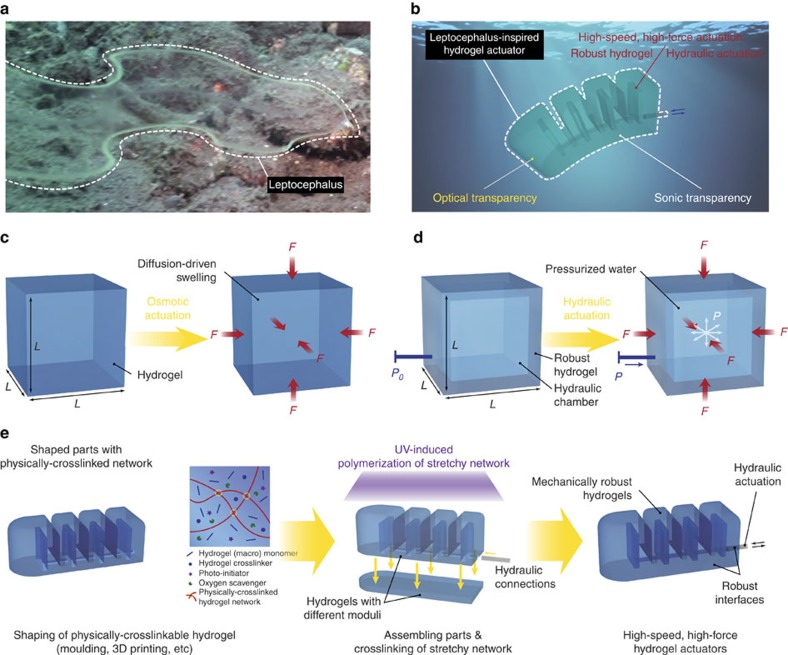
Design and fabrication of leptocephalus-inspired hydrogel actuators. (**a**) Leptocephalus in oceanic environment (the image is credited to HRF U/W Production). (**b**) Schematic illustration of leptocephalus-inspired hydrogel actuators capable of high-speed, high-force actuation with optical and sonic transparency in water. (**c**) Schematic illustration of osmotic-driven actuation of hydrogels. (**d**) Schematic illustration of hydraulic-driven actuation of hydrogels. (**e**) Schematic illustration of the fabrication of complex and robust hydrogel structures for hydraulic hydrogel actuators.

**Figure 2:**
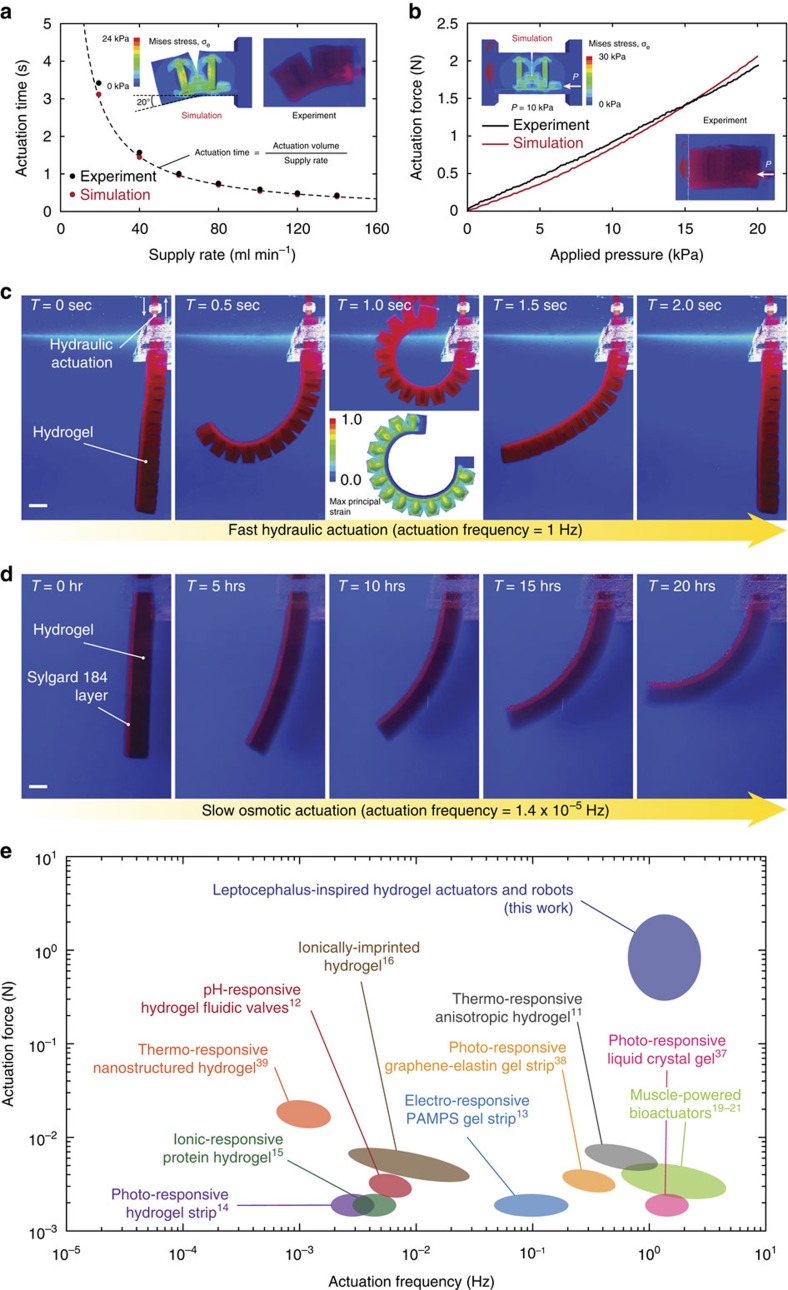
Hydraulic actuation of hydrogel in water. (**a**) Actuation time is defined as a ratio of actuation volume to supply rate. Actuation time versus supply rate from the pump for the unit-segment hydraulic hydrogel actuator in both experiments and finite element simulations. (**b**) Actuation force versus applied pressure for the unit-segment hydraulic hydrogel actuator in both experiments and finite element simulations. (**c**) Fast bending actuation of the hydraulic hydrogel actuator with actuation frequency around 1 Hz. The finite element simulation also captures the fully actuated state of the actuator and its maximum principal strain (middle inset image). (**d**) Slow swelling-driven osmotic actuation of a bulk PAAm-alginate hydrogel assembled with a Sylgard 184 layer. (**e**) Comparison of the actuation forces and frequencies between the hydraulic hydrogel actuators in this study and typical osmotic hydrogel actuators and muscle-powered bioactuators^11^,^12^,^13^,^14^,^15^,^16^,^19^,^20^,^21^,^37^,^38^,^39^. Note that the hydrogel actuators in **a**–**d** are dyed with colour for better visual representation. Scale bars, 1 cm (**c**,**d**).

While the active and transparent hydrogel-based tissues and organs of leptocephali endow them with agile motion and natural camouflage in water, such striking capabilities have not been achieved by synthetic hydrogel actuators and robots (Fig. 1a,b and Supplementary Movie 1)^1^,^2^. Despite recent developments of hydrogels responsive to various stimuli^11^,^12^,^13^,^14^,^15^,^16^, existing hydrogel actuators are mostly osmotic-driven (that is, based on swelling/de-swelling) and suffer from the intrinsic coupling between responsive times and the actuation forces. As illustrated in Fig. 1c, the swelling-induced force *F* from a constrained hydrogel block and the responsive time *t* scale as


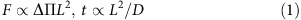


where ΔΠ is the osmotic-pressure change, *D* diffusivity of water in the hydrogel, and *L* a typical dimension of the block. With the same osmotic-pressure change, a higher actuation force requires a larger size of the hydrogel block, which leads to longer responsive time. As a result, osmotic-driven hydrogel actuators are generally limited by low actuation speeds (for example, responsive time of minutes to hours) and/or low actuation forces (for example, millinewtons) (Fig. 2e).

To overcome the force-time coupling of osmotic hydrogel actuators, we propose to exploit hydraulic actuations of hydrogel structures to achieve much higher actuation forces and speeds than existing osmotic hydrogel actuators^11^,^12^,^13^,^14^,^15^,^16^ and muscle-powered bioactuators^19^,^20^,^21^. As illustrated in Fig. 1d, the hydraulic-induced actuation force *F* from a constrained hydrogel block and the responsive time *t* scale as


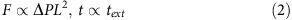


where Δ*P* is the applied pressure in the hydraulic chamber of the hydrogel, and *t*_*ext*_ the responsive time of external system such as a pump to supply the pressure. Since the actuation force and speed are de-coupled in hydraulic hydrogel actuators (equation (2)), both can achieve higher values than osmotic hydrogel actuators (Fig. 2e)^22^,^23^. Furthermore, because water is pumped in and out of the hydraulic chambers in hydrogels, the actuation does not affect optical or sonic transparency of the hydrogels in water, unlike osmotic hydrogel actuators that often turn opaque in de-swollen states.

### Fabrication of hydraulic hydrogel actuators and robots

Whereas hydraulic soft actuators are mostly fabricated with flexible silicone elastomers (for example, Ecoflex, Elastosil and Sylgard 184) (refs 4, 22, 24) and their developments have been well documented in online resources (for example, softroboticstoolkit.com), it has not been possible to make hydrogels into hydraulic actuators or robotic structures. One central challenge is to fabricate hydrogel structures that maintain robustness and functionality under cyclic hydraulic actuations without leakage or failure from the bodies and interfaces. Here we invent a simple method to fabricate transparent and robust hydrogel structures capable of sustaining multiple cycles of hydraulic actuations (for example, over 1,000 cycles). The method takes advantage of the physical and chemical hybrid crosslinks in tough hydrogels and the hydrogels' capabilities of robust adhesion on various materials^25^,^26^,^27^,^28^. As many tough hydrogels are composed of physically crosslinked dissipative polymer networks and covalently crosslinked stretchy polymer networks^25^,^26^,^27^,^28^, simple parts of complicated hydrogel structures can be first moulded or 3D printed by physically crosslinking the dissipative networks in pre-gel solutions (Fig. 1e). Thereafter, the shaped parts are assembled into the designed hydrogel structures, followed by covalently crosslinking the stretchy networks inside the parts and across interfaces between different parts (Fig. 1e). Other materials (for example, elastomeric tubing) can also be moulded or assembled with the hydrogel parts, and then covalent grafting of stretchy networks in the hydrogels on other materials' surfaces gives robust adhesion between hydrogels and other materials^26^,^27^. The resultant hydrogel actuators are immersed in water for 48 h to allow them to reach equilibrium swollen states, while the bodies and the interfaces in the hydrogel actuators maintain high robustness (Supplementary Fig. 1). Using the proposed method, we fabricate a set of hydraulic hydrogel actuators and robots based on polyacrylamide (PAAm)-alginate tough hydrogels with different moduli^22^,^24^ (see Methods and Supplementary Figs 2 and 3 for details on fabrication of the actuators). Notably, the method is also applicable to other tough hydrogels with physical and chemical hybrid crosslinks^27^,^28^.

### Hydraulic actuation of hydrogel actuators and robots

**Figure 3:**
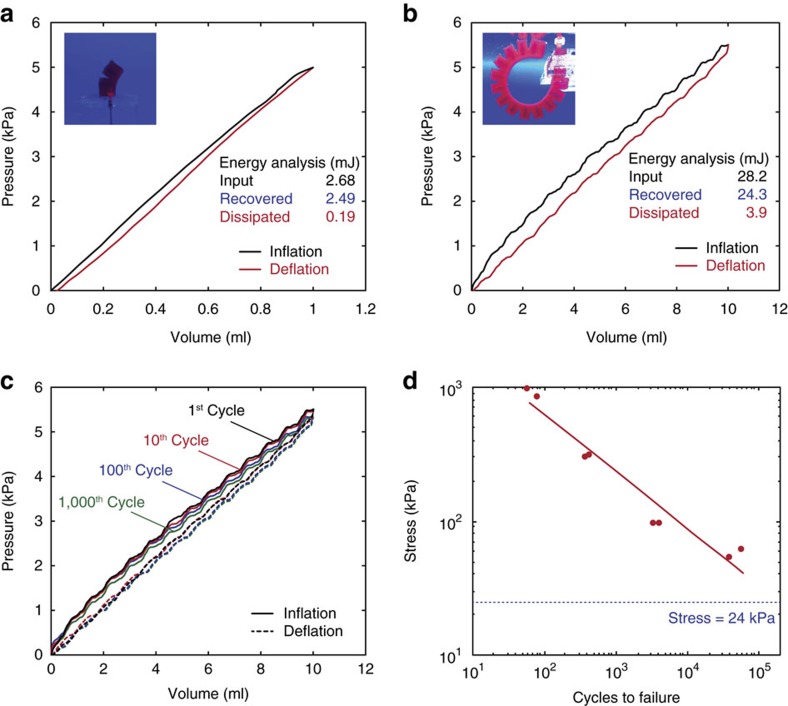
Characterization of the hydraulic hydrogel actuators under cyclic actuations. (**a**) Pressure-volume hysteresis curve and the resultant energy analysis for the unit-segment hydrogel actuator. (**b**) Pressure-volume hysteresis curve and the resultant energy analysis for the bending hydrogel actuator. (**c**) Pressure-volume hysteresis curves for the bending hydrogel actuator in the 1st, 10th, 100th and 1,000th cycles of actuations with the actuation frequency of 0.5 Hz. (**d**) The number of cycles to failure for the PAAm-alginate hydrogel versus the applied nominal stress. The cyclic fatigue tests are performed using stress-controlled cyclic tension of the hydrogel samples at a frequency of 1 Hz. The dotted line indicates the maximum stress level in the fully actuated hydrogel actuator in Fig. 2a. Note that hydrogel actuators in **a**,**b** are dyed with colour for better visual representation.

To evaluate the performance of hydraulic hydrogel actuators, we first measure the actuation speed and force of a unit-segment hydrogel actuator with dimensions of 15 × 25 × 40 mm that consists of two hydraulic chambers (Supplementary Fig. 2a). As shown in Fig. 2a, this macroscale hydrogel actuator can give impressively fast motion (that is, bending of 20 degrees in less than 1 s), where the actuation time is equal to the volume of water supplied for one actuation divided by the external supply rate (Fig. 3a, Supplementary Fig. 4a and Supplementary Movie 2). The actuation force generated by a constrained actuator scales approximately linearly with the externally applied hydraulic pressure (equation (2)) and can reach over 2 N under a hydraulic pressure of 20 kPa (Fig. 2b and Supplementary Fig. 5). The maximum hydraulic pressure is determined by mechanical properties of constituent hydrogels and the structure of hydraulic actuators, which can undergo unstable inflation (for example, bulging) under excessively high applied pressure (Supplementary Fig. 4b). Notably, although the pressure difference inside and outside the hydraulic chambers can cause water diffusion through hydrogel structures, such effect is negligible over the time scale (that is, seconds) of hydraulic actuation (see Methods for detailed discussion). In addition, the experimental results match consistently with predictions from finite element models (Fig. 2a,b), which can be used to guide the design and optimization of future hydrogel actuators and robots.

In Fig. 2c, we demonstrate the fast actuation of a more complicated hydraulic hydrogel actuator that consists of seven serially connected unit-segment actuators. As water is pumped into the actuation chambers, the originally straight hydrogel actuator bends into a full circle within 1 s; when the water is pumped out, the actuator restores its straight shape within 1 s (Figs 2c and 3b and Supplementary Movies 3 and 4). In contrast, an osmotic hydrogel actuator with similar dimensions and made of the same hydrogel as the hydraulic one takes 20 h to deform into half a circle (Fig. 2d and Supplementary Movie 5). Based on equation (1), for the osmotic hydrogel actuator to reach an actuation time of seconds, its dimension needs to reduce from centimeters to less than 100 μm, which will reduce the actuation forces from newtons to less than 1 mN. This dramatic contrast clearly demonstrates the much higher actuation speed and/or force of hydraulic hydrogel actuators than the corresponding osmotic hydrogel actuators (Fig. 2d and Supplementary Movie 5). From a more comprehensive comparison in Fig. 2e, we can further see that the actuation force and/or actuation frequency (that is, inverse of actuation time) of the hydraulic hydrogel actuators can be orders of magnitude higher than the typical performance of osmotic hydrogel actuators responsive to pH, temperature, electric fields, ionic strength and light and muscle-powered bioactuators^11^,^12^,^13^,^14^,^15^,^16^,^19^,^20^,^21^. (Note that the comparison on actuation force and speed in Fig. 2e is made between hydraulic and osmotic hydrogel actuators not with elastomer-based soft actuators and robots.) It is also expected that the proposed hydrogel actuators and robots can operate in air with pneumatic actuation instead of hydraulic actuation.

In addition, the hydraulic hydrogel actuators can maintain robustness and functionality over multiple cycles of actuations without failure or leakage. In Fig. 3c, we show that the bending actuator can withstand over 1,000 continuous cycles of fast actuations (for example, 0.5 Hz actuation frequency) without failure of the body or the interfaces. The hydrogel actuator also maintains its performance over multiple cycles as its pressure-volume hysteresis curves are kept in the similar level up to 1,000 cycles (Fig. 3c). The hydrogel actuator exhibits very slight decrease in the maximum actuation pressure over cycles possibly due to softening of PAAm-alginate tough hydrogel under cyclic deformation^25^,^26^. To further understand the cyclic behaviour of the hydrogel actuators, we carry out fatigue tests on the PAAm-alginate hydrogel. The hydrogel samples are cyclically loaded to different levels of applied nominal stress (that is, applied force over undeformed cross-section area of the sample) until failure (Supplementary Fig. 6). We plot the applied nominal stress versus the number of cycles to failure in Fig. 3d. It can be seen that the PAAm-alginate hydrogel possesses good anti-fatigue properties under low and moderate levels of applied stress. For example, an applied stress of 24 kPa, which is the maximum stress in the fully actuated hydrogel actuator in Fig. 2a, will not cause fatigue failure of the hydrogel over 40,000 cycles (Fig. 3d). This anti-fatigue property of PAAm-alginate hydrogel supports the robustness of hydraulic hydrogel actuators and robots for long-term applications (Fig. 3d).

### Optical and sonic transparency in water

**Figure 4:**
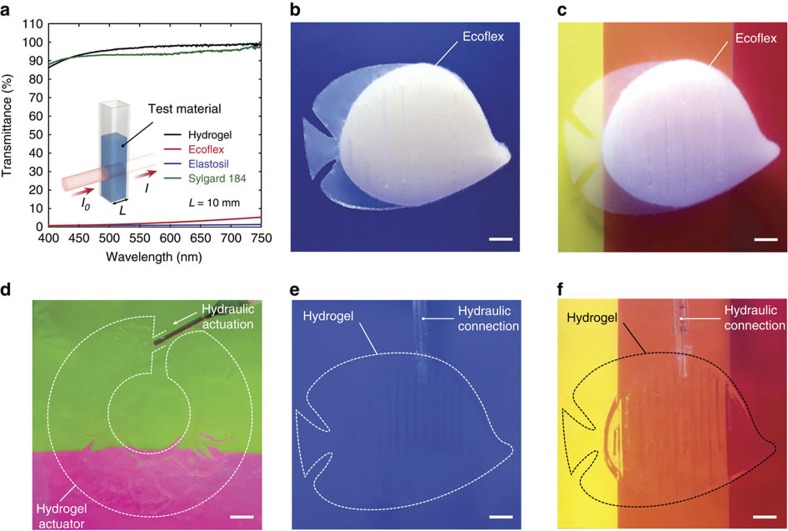
Optically camouflaged hydrogel actuators and robots in water. (**a**) Transmittance in visible light range for the PAAm-alginate hydrogel, Ecoflex, Elastosil and Sylgard 184. (**b**,**c**) Images of the hydraulic fish-like actuators made of Ecoflex in mono-colour (**b**) and multi-colour (**c**) backgrounds. (**d**) Image of the fully actuated bending hydraulic hydrogel actuator in multi-colour background. (**e**,**f**) Images of the hydraulic fish-like actuators made of PAAm-alginate hydrogel in mono-colour (**d**) and multi-colour (**e**) backgrounds. Dotted lines are introduced in **d**–**f** to indicate the boundaries of transparent hydrogel structures in water. Scale bars, 1 cm (**b**–**f**).

**Figure 5:**
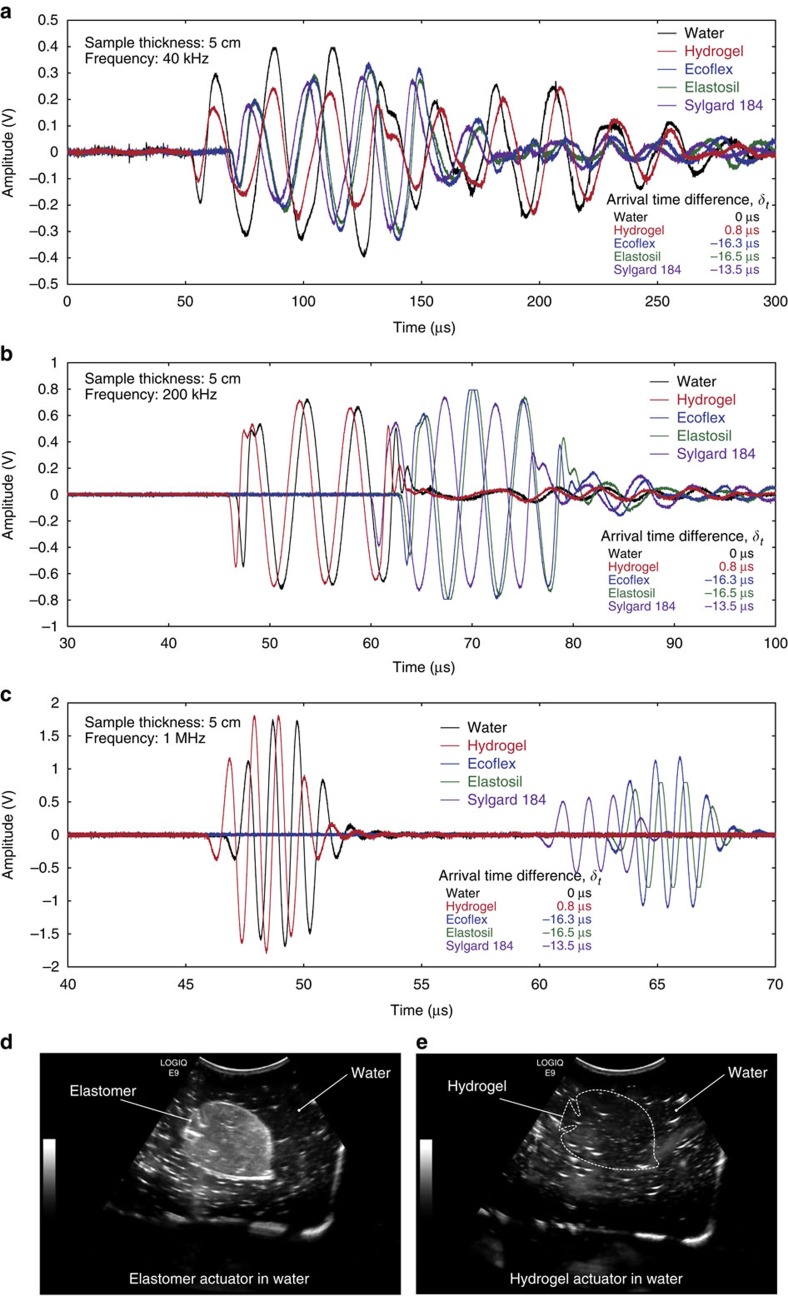
Sonically camouflaged hydrogel actuators and robots in water. (**a**–**c**) Speed of sound measurements for pure water, PAAm-alginate hydrogel, Ecoflex, Elastosil and Sylgard 184. The curves indicate the ultrasound signals travel through the samples from the transducer to the hydrophone with the source frequency of 40 kHz (**a**), 200 kHz (**b**) and 1 MHz (**c**). The *t*=0 corresponded to the time at which the ultrasound signal was sent from the transducer and the signal amplitudes were measured by the hydrophone upon the arrival of the transmitted ultrasound signals through the samples. The ultrasound signals sent at each frequency had the same amplitude while the attenuation varied among sample materials due to different acoustic impedance and viscous effect of each material. (**d**,**e**) Ultrasound image of the hydraulic fish-like actuators made of Ecoflex (**d**) and PAAm-alginate hydrogel (**e**) in the water tank. Dotted lines are introduced in **e** to indicate the boundaries of transparent hydrogel structures in water.

We next exploit the high optical and sonic transparency of hydraulic hydrogel actuators in water to achieve leptocephali-like natural camouflage in both optical and sonic environments. The PAAm-alginate hydrogels used in the current study contain more than 90 wt.% of water^25^,^26^,^27^,^28^, and the hydraulic chambers are also filled with water. This high water content in the hydraulic hydrogel actuators endows them with optical and sonic properties almost the same as those of water (Figs 4 and 5, and Table 1)^1^,^25^,^29^. To make comparison on optical and sonic properties between the PAAm-alginate hydrogels and commonly used elastomers for soft actuators and robots, three most frequently used silicone elastomers (that is, Ecoflex, Elastosil and Sylgard 184) in the soft actuators and robots literatures are selected in this study^4^,^22^,^23^,^24^ (Table 1). As shown in Fig. 4a and Table 1, the transmittance of a piece of PAAm-alginate hydrogel with thickness of 10 mm is∼95% in visible light range, and its reflective index is 1.3365, only around 0.3% different from the reflective index of water. In contrast, highly stretchable silicone elastomers like Ecoflex and Elastosil for soft actuators and robots are optically opaque, and the transmittance of the elastomers with thickness of 10 mm is less than 5% in visible light range (Fig. 4a and Table 1). Although Sylgard 184 exhibits high transparency with over 90% transmittance, it usually needs to be used together with other stretchable yet less transparent elastomers (that is, Ecoflex or Elastosil) in soft actuators or robots, due to the brittleness and low stretchability of Sylgard 184 (refs 4, 5, 22).

In order to quantify the sonic properties, we measure the speed of sound in the PAAm-alginate hydrogel and elastomers over three different frequencies (that is, 40 kHz, 200 kHz and 1 MHz), representing the frequencies used in various applications (Fig. 5a–c). For instance, the low-frequency ultrasound (10∼50 kHz) is frequently used for long-range SONAR or oceanic animal communications while the mid-frequency ultrasound (100∼300 kHz) is usually used for high-resolution SONAR or by some animals (for example, dolphins)^30^. For medical diagnostic ultrasound, higher frequencies (1∼5 MHz) are usually adopted^31^. Notably, the measured speed of sound is identical over these selected frequencies without dispersion for all sample materials (Fig. 5a–c) (see Methods for details on measurements). We found that the PAAm-alginate hydrogel shows acoustic impedance, *z*_0_=1.487 × 10^6^ Pa s m^−1^, which is only around 1% different from the acoustic impedance of pure water (that is, *z*_0_=1.448 × 10^6^ Pa s  m^−1^) as the speed of sound inside the hydrogel and pure water and their density are nearly identical (Fig. 5a–c and Table 1) (see Methods for detailed discussion). As a result, the acoustic reflection coefficient between water and the hydrogel is as low as *R*=0.013. In contrast, elastomers have much lower characteristic acoustic impedances than water (that is, *z*_0_=1.052 × 10^6^ Pa s m^−1^ for Ecoflex, *z*_0_=1.058 × 10^6^ Pa s m^−1^ for Elastosil and *z*_0_=1.053 × 10^6^ Pa s m^−1^ for Sylgard 184) regardless of their optical properties leading to much higher acoustic reflection coefficients against water than hydrogels (that is, *R*=0.158 for Ecoflex, *R*=0.156 for Elastosil and *R*=0.158 for Sylgard 184) (Fig. 5a–c and Table 1).

As optical light and reflective sonic waves are two most commonly used sensing strategies by biological and engineered systems in underwater environments, the close similarities in optical and sonic properties between water and hydrogels make the hydraulic hydrogel actuators optically and sonically transparent in water, enabling leptocephali-like passive camouflage (Figs 4 and 5, and Supplementary Fig. 7a). In Fig. 4b–f, we place hydraulic fish-like actuators with the same dimensions but made of Ecoflex or hydrogels in water tanks against different background colours. The fish-like actuators made of Ecoflex are clearly visible in front of monocolour (Fig. 4b) and multicolour (Fig. 4c) backgrounds. In contrast, the hydrogel fishes with the same structure are optically camouflaged in both backgrounds (Fig. 4e,f). Moreover, the bending hydrogel actuator in Fig. 2c (without dye) can be fully actuated without losing its optical transparency against a multicolour background (Fig. 4d). The sonic camouflage of the hydraulic hydrogel actuators in water is remarkable as well. We take ultrasound images of hydraulic fish-like actuators made of Ecoflex or hydrogels in water tanks (Fig. 5d,e). (Note that the speckle patterns in the ultrasound images come from reflected sound waves against the wall of the water tank.) Owing to very low acoustic reflection coefficient between water and hydrogels (that is, *R*=0.013), the hydrogel fish is undistinguishable from surrounding water in the ultrasound image (Fig. 5e and Supplementary Fig. 7a). In contrast, the same structure made of Ecoflex is clearly visible against surrounding water in the ultrasound image, owing to much higher acoustical reflection for elastomers (Fig. 5d and Supplementary Fig. 7a).

### Applications of hydraulic hydrogel actuators and robots

**Figure 6:**
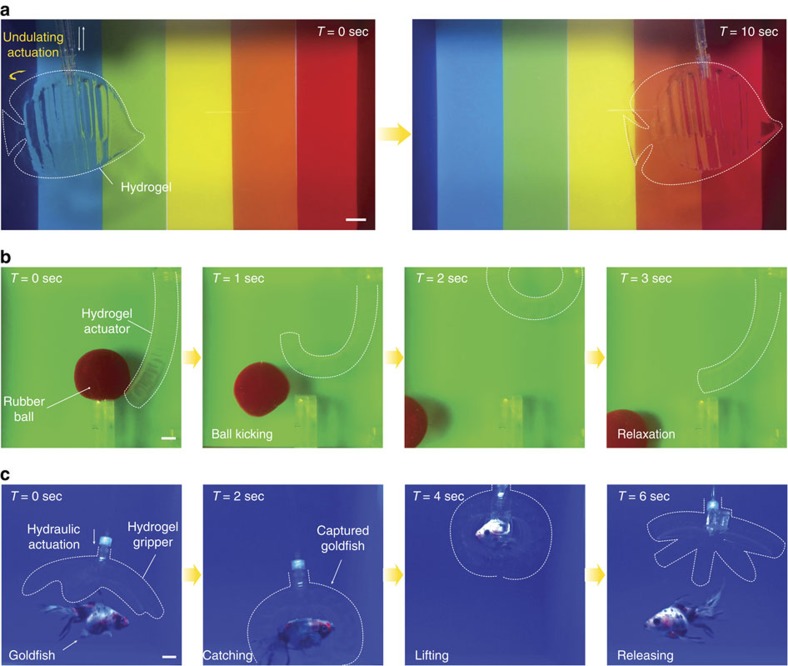
Various applications of naturally camouflaged hydrogel actuators and robots. (**a**) Forward fish-like swimming of a hydrogel robotic fish in water. The hydrogel fish can keep the camouflaged state when swimming over rainbow-coloured background owing to its optical transparency. (**b**) A transparent hydrogel actuator kicks a rubber-ball in water. The high-speed and high-force hydraulic actuation enables effective ball-kicking motion. (**c**) A transparent hydrogel gripper catches, lifts and releases a live ryukin goldfish. The agile actuation and optical transparency of the hydrogel gripper allow its successful capture of the goldfish. The gripper holds and then releases the captured goldfish without harm owing to the gripper's softness. Dotted lines are introduced in **a**–**c** to indicate the boundaries of transparent hydrogel structures in water. Scale bars, 1 cm (**a**–**c**).

We further demonstrate a few functions and applications uniquely enabled by the agile hydrogel soft actuators and robots naturally camouflaged in water (Fig. 6 and Supplementary Fig. 8). The hydrogel robotic fish is not only invisible in water but can generate agile tail actuations (that is, 1 s per stroke). The hydraulic-driven fast actuation can successfully achieve forward fishlike locomotion, while maintaining the camouflaged state during swimming over a rainbow-coloured background (Fig. 6a and Supplementary Movie 6) (see Methods and Supplementary Fig. 8a for details on the experimental setup). As another example, the agile and forceful motion of the bending actuator can apply forces and do works on various objects under water. In Fig. 6b and Supplementary Movie 7, we show that the hydrogel actuator can kick off a rubber-ball with diameter of 7.5 cm in water within 1 s, and the actuator maintains the camouflaged state against different backgrounds during the motion. In addition, a naturally camouflaged hydrogel gripper can even catch and release a live goldfish in water (Fig. 6c and Supplementary Movie 8). Optical transparency of the hydrogel gripper keeps its camouflaged state when it approaches the proximity of the goldfish in the water tank while its agile actuation allows successful catching and gripping motion (Fig. 6c). In addition, the hydrogel actuators' intrinsic softness enables release of the caught goldfish without causing any harm to the goldfish (Fig. 6c).

## Discussion

We have demonstrated simple design, fabrication and operation of hydraulic-driven hydrogel actuators and robots that give high-speed (that is, responsive time less than 1 s), high-force (that is, over 1 N) actuations compared to osmotic hydrogel actuators and are capable of optical and sonic camouflage in underwater environments. The hydrogel actuators and robots can maintain their robustness and functionality over multiple cycles (that is, over 1,000) of actuations, due to the anti-fatigue property of the PAAm-alginate hydrogel under moderate stresses. It is also expected that pneumatic actuation can be used to drive the hydrogel actuators and robots to operate in air. The technology and systems developed here may enable new opportunities in diverse scientific and engineering fields. The principle and fabrication method to decouple actuation force and speed in hydrogel actuators can guide the design of next-generation responsive hydrogels. Soft, agile and powerful devices made of hydrogels can give more comfortable and biocompatible handling and/or stimulation of biological organisms^3^; while the hydrogels' optical and sonic transparency will allow real-time high-fidelity optical and ultrasound imaging through the devices. Bioactive components (for example, drugs, bacteria and mammalian cells) can be further incorporated in the hydrogel devices for sustained release and controlled delivery^32^. Marine biologists can use hydrogels to design next-generation biomimetic robots more realistic than elastomeric and metallic ones to study their interactions with sea animals^1^,^2^. Leptocephali-inspired camouflage endowed by hydrogels' optical and sonic camouflage may find critical applications that require prolonged and passive avoidance of detection such as robots for underwater reconnaissance.

## Methods

### Materials

Acrylamide (AAm; Sigma-Aldrich A8887) was used as the monomer for the covalently crosslinked stretchy network in the tough hydrogel. In the polyacrylamide (PAAm) network, N,N-methylenebisacrylamide (MBAA; Sigma-Aldrich 146072) was used as crosslinker and 2-hydroxy-4′-(2-hydroxyethoxy)-2-methylpropiophenone (Irgacure 2959; Sigma-Aldrich 410896) was used as photoinitiator. Sodium alginate (Sigma-Aldrich A2033) ionically crosslinked with calcium sulfate (Sigma-Alginate C3771) was used for the physically crosslinked dissipative network in the tough hydrogel. To alleviate the oxygen inhibition during moulding process, glucose (Sigma-Aldrich G8270) and glucose oxidase (Sigma-Aldrich G7141) were added in to pre-gel solution as an oxygen scavenger. For surface treatment of elastomeric hydraulic connections, benzophenone (Sigma-Aldrich B9300) was used. As hydraulic connections, various sizes of silicone tubings (McMaster Carr) and metallic needles (Nordson EFD) were used. To colourize hydrogels for better visual representation, red food dye (McCormick) was used. For elastomers in experiments, Ecoflex 30 (Smooth-On), Elastosil M 4601 (WACKER Chemical) and Sylgard 184 (Dow Corning) were used.

### Fabrication of hydraulic hydrogel actuators

The hydraulic hydrogel actuators were designed based on previously reported soft actuators (Supplementary Fig. 2)^4^,^5^,^22^,^24^. The moulds for each parts were fabricated using 3D printer (Form2; Formlabs) and laser cutter (Epilog Mini/Helix; Epilog Laser) based on computer-aided design drawings generated from commercial 3D modelling software (Solidworks; Dassault Systems). Each hydrogel parts were prepared by physically crosslinking hydrogels using the moulds. Physically crosslinked hydrogels were fabricated by pouring pre-gel solution into the moulds and covering glass plates. The pre-gel solution for the soft PAAm-alginate tough hydrogel was prepared by mixing carefully degassed aqueous solution (22.2 wt.% AAm, 1.5 wt.% sodium alginate, 0.02 wt.% MBAA, 0.2 wt.% Irgacure 2959, 2.2 wt.% glucose and 0.01 wt.% glucose oxidase) with ionic crosslinkers (calcium sulfate slurry with final concentration of 20 × 10^−3^ M in crosslinked hydrogel). The pre-gel solution for the stiff PAAm-alginate tough hydrogel was prepared by mixing carefully degassed aqueous solution (22.2 wt.% AAm, 3 wt.% sodium alginate, 0.02 wt.% MBAA, 0.2 wt.% Irgacure 2959, 2.2 wt.% glucose and 0.01 wt.% glucose oxidase) with ionic crosslinkers (calcium sulfate slurry with final concentration of 60 × 10^−3^ M in crosslinked hydrogel). The physically crosslinked hydrogels in the moulds were kept in a humid chamber for 1 h to make sure the formation of physical crosslinks. Thereafter, the physically crosslinked hydrogel parts were carefully separated from the moulds and assembled each other together with hydraulic connections (for example, elastomeric tubings). Elastomeric tubings were treated with benzophenone solution (10 wt.% in ethanol) for 2 min at the room temperature (23 °C) to form robust interface with hydrogel^27^. Treated elastomeric tubings were washed with methanol three times and completely dried with nitrogen gas before being assembled with the physically crosslinked hydrogel parts.

The assembled parts were further exposed to UV-irradiation in a UV chamber (365 nm UV; UVP CL-1000) for an hour to form covalently crosslinked stretchy polyacrylamide networks. Note that the UV chamber was kept humid to avoid potential drying of hydrogels during the second crosslinking process. After the polymerization of covalently crosslinked polyacrylamide networks in the tough hydrogels, the assembled hydrogel parts and hydraulic connections form robust interfaces^26^,^27^. Before being used in experiments and measurements, the fabricated hydraulic hydrogel actuators and robots were immersed in deionized water at least for 48 h to reach equilibrium swollen state. Note that the degree of swelling for both soft and stiff PAAm-alginate tough hydrogels were kept in the similar level (for example, equilibrium volumetric swelling ratio∼2.5) to avoid distortion in geometry after swelling. The equilibrium water contents of the fully swollen PAAm-alginate tough hydrogels was around 94 wt.%.

### Measurement of interfacial strength between assembled hydrogels

To measure interfacial toughness of the assembled hydrogels, two types of samples—tough hydrogels prepared as a whole part and tough hydrogels prepared by assembling two parts—were used. The final size of the samples was fixed at 15 mm in width, 100 mm in length and 3 mm in thickness. For tough hydrogels prepared as whole part, the whole sample was fabricated using soft PAAm-alginate tough hydrogel without assembly process. For tough hydrogels prepared by assembly, two separately prepared physically crosslinked soft PAAm-alginate tough hydrogels with 15 mm in width, 100 mm in length and 1.5 mm in thickness were assembled together following the proposed method giving the same final size. One side of all samples was strongly adhered onto the surface treated glass plates by the previously reported method^26^. As a stiff backing for the hydrogel, thin polyethylene terephthalate (PETE) film (70 μm thickness) was introduced onto the hydrogel with cyanoacrylate adhesive. Before measurements, all samples were immersed in deionized water for 48 h to reach equilibrium swollen-state. To clearly measure the interfacial toughness between the assembled hydrogels, 1 cm length notch was introduced at the interface between the assembled hydrogels or at the same location for the tough hydrogels prepared without assembly. Then, the fully swollen samples were tested with the standard 90-degree peeling test (ASTM D 2861) with mechanical testing machine (20 N load cells; Zwick/Roell Z2.5) and 90-degree peeling fixture (G50; Test Resources) under a constant cross-head speed of 50 mm min^−1^(Supplementary Fig. 1a). As bulk hydrogel or assembled hydrogels were separated during the tests, the measured force reached a plateau (with slight oscillations), as the separation process entered steady-state (Supplementary Fig. 1b). The interfacial toughness was determined by dividing the plateau force *F* by the width of the hydrogel sample *W* (Supplementary Fig. 1c)^26^,^27^. Notably, the measured interfacial toughness was similarly high (over 1,500 J m^−2^) for both types of samples indicating that the interfacial strength of the assembled hydrogels is as robust as the bulk tough hydrogel.

### Hydraulic actuation of hydrogel actuators

Hydraulic hydrogel actuators were actuated by pressurized water supplied from multiple programmable high-throughput syringe pumps (New Era Pump Systems, Inc.). Pressurized water from the syringe pumps was supplied through hydraulic connections (for example, silicone tubings and metallic needles) into the hydrogel actuators. The inflation and deflation of actuators were realized by infusion and withdrawal of water by the syringe pumps that were programmed by custom codes. The actuation speed was controlled by programming the syringe pumps with appropriated supply flow rates. Synchronous actuation of two sides of the hydrogel fish was realized by adopting two separately controlled sets of syringe pumps (Supplementary Fig. 8a). Air bubbles within hydraulic chambers of hydrogel actuators were completely removed by squeezing or degassing hydrogel actuators inside water.

Hydrogels are permeable to water due to diffusive transportation of water through hydrogels. The steady-state diffusive transportation of water through hydrogel structures follows the relation^33^,


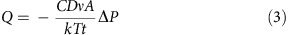


where *Q* represents flow rate of permeated water through the hydrogel, *C* represents volume concentration of water molecules in the hydrogel, *D* represents the diffusivity of water molecule in the hydrogel, *v* represents the volume per water molecule with the typical representative value of 10^−28^ m^3^, *A* represents the surface area of the hydrogel, *k* represents the Boltzmann constant, *T* represents the absolute temperature, *t* represents thickness of the hydrogel wall of the chamber, and Δ*P* represents the pressure gradient applied to the hydrogel hydraulic chambers. Typical values of diffusivity, *D* of the fully swollen PAAm-alginate tough hydrogel is in the order of 3 × 10^−10^ m^2^ s^−1^ (ref. 28). Also, the representative values of the area *A* and the thickness *t* for the unit-segment hydrogel actuator are 10 cm^2^ and 3 mm, respectively. The value of *kT* at the room temperature (23 °C) is 4 × 10^−21 ^J. The water concentration *C* is around 90% and the hydraulic hydrogel actuators were actuated with the applied pressure gradient of 5 kPa (Fig. 3a,b). Therefore, the typical value of permeated water flow rate, *Q* for the unit-segment hydrogel actuator is 1.1 × 10^−8^ ml s^−1^ which is several orders of magnitudes smaller than the typical supply flow rate from the pumps (for example, 2 ml s^−1^ at 2 Hz actuation frequency). As water permeation through the hydraulic hydrogel chambers is much smaller than the typical supply flow rate, the effect of water permeation is negligible over the time scale (that is, seconds) of hydraulic actuation.

### Finite element simulation of hydraulic hydrogel actuators

To investigate the strain and stress distribution and the actuation time and force of hydraulic hydrogel actuators, we built 3D finite element models using a commercial finite element software ABAQUS 6.14 based on the previously reported approaches^24^,^34^,^35^. The geometry of the actuator was imported into ABAQUS CAE using STEP files generated from commercial 3D modelling software (Solidworks; Dassault Systems). The imported geometry was subsequently meshed using solid quadratic tetrahedral elements (ABAQUS element type C3D10M) for both hydraulic chambers and a stiff layer that are modelled as hydrogels with different mechanical properties (that is, soft and stiff tough hydrogels) (Supplementary Fig. 3). To capture the elastic response of the actuators, we modelled both hydraulic chambers and stiff layer as hyper-elastic solid using incompressible Ogden model whose strain energy density is given by


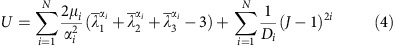


where 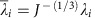, *μ*_*i*_ and *α*_*i*_ represent material parameters fitted from the experimentally measured data, and *N* represents the order parameter which is set as 1 here. By fitting with the measured stress-strain curve, the material parameters for hydraulic chamber were identified as *μ*_1_=6.17895, kPa, *α*_1_=3.78464 while that for stiff layer was fitted as *μ*_1_=27.92793, kPa, *α*_1_=2.52527 (Supplementary Fig. 3). Note that both stiff and soft PAAm-alginate tough hydrogels were deformed up to the maximum deformation in the actual actuators and robots in this study (that is, ∼1.2 for the stiff tough hydrogel and ∼2.7 for the soft tough hydrogel).

Thereafter, dynamic explicit simulations were performed by applying flux of incompressible water on enclosed faces of the chambers and constrained one side of the actuator to simulate the experimental boundary conditions. To impose the incompressible condition of hydrogel materials, we set the Poisson's ratio as high as 0.49. Mass scaling technique was adopted to maintain a quasi-static process during the actuating process. The actuation speed was determined by checking the time to reach a fully actuated state (for example, bending of 20 degrees for the unit-segment actuator) under the prescribed flux on the internal faces of the chamber (Fig. 2a and Supplementary Fig. 4a). To further predict the force generated by the unit-segment actuator, we prescribed the flux on the internal faces of the chamber with both sides constrained (Fig. 2b and Supplementary Fig. 5b). As hydrogel hydraulic actuators were operated in water and had density nearly the same as water in the fully swollen state, gravitational forces were not taken into account in finite element simulations.

### Measurement of actuation force and speed

The actuation time was defined as the time required to reach the fully actuated state (for example, bending of 20 degrees for the unit-segment hydrogel actuator). The actuation time was determined using the real-time videos of hydraulic actuations under varying supply flow rates (Fig. 2a). The actuation volume (that is, required volume of water to generate full actuation) of the unit-segment hydrogel actuator was found to be 1 ml (Fig. 3a). The supply flow rate of 120 ml min^−1^ was used to generate actuation frequency (that is, inverse of actuation time) of 2 Hz for the unit-segment actuator (Supplementary Fig. 4a). The full actuation for the bending hydrogel actuator was defined as touching of the fixed end by its free end, and its actuation volume was found to be 10 ml (Fig. 3b). The supply flow rate of 600 ml min^−1^ was used to generate actuation frequency of 1 Hz for the bending actuator (Fig. 2c). To measure the force generated by the unit-segment hydrogel actuator, the actuator was placed between a rigid bottom plate and an acrylic fixture connected to a force sensor (NeuLog; Fisher Scientific) (Supplementary Fig. 5a). Then, water was slowly infused into the actuator with the supply flow rate of 1 ml min^−1^ by the syringe pump while the pressure within the actuator was recorded by a pressure sensor (PX 409; Omega Engineering Inc.) connected via T-junction. The sufficiently low infusion rate was adopted to maintain quasi-static conditions. The actuation force was recorded by the force sensor, and then plotted against the recorded pressure (Fig. 2b). The measurement was stopped at the internal pressure around 20 kPa as the unit-segment hydrogel actuator underwent unstable inflation (for example, bulging) for higher pressure level (Supplementary Fig. 4b).

### Pressure-volume hysteresis curves

To analyse mass transport characteristics of the hydraulic hydrogel actuators, pressure-volume hysteresis curves were generated by hydraulic inflation and deflation of the actuators in water following the previously reported methodology^24^. The syringe pump was connected to the hydrogel actuators that were clamped and submerged in water in a vertical position, and the pressure sensor was also connected via T-junction. Water was infused into the hydrogel actuator with sufficiently low supply flow rate (for example, 5 ml min^−1^) until the amount of supplied water reached the actuation volume (for example, 1 ml for the unit-segment actuator and 10 ml for the full-bending actuator), then the actuator was deflated by withdrawing water from it. Once the actuator had completed one full cycle and the pressure returned to zero, the same cycle was repeated at least three times to assure reproducibility. The recorded pressure was plotted against volume to form pressure-volume hysteresis curves and energy analysis was performed using the area under the pressure-volume hysteresis curves (Fig. 3a,b).

To further investigate the reliability of the hydraulic hydrogel actuators under cyclic actuations, we performed cyclic fatigue tests of the hydrogel actuators. The bending actuators were actuated by the programmed syringe pumps more than 1,000 continuous inflation-deflation cycles with actuation frequency of 0.5 Hz (300 ml min^−1^ supply flow rate). At each 1st, 10th, 100th and 1,000th cycle, pressure-volume hysteresis curves were obtained for comparison by the procedure described above (Fig. 3c). After 1,000 cycles, the hydrogel actuator did not show neither material failure nor interfacial failure.

### Cyclic fatigue failure characterization of the hydrogel

To characterize the cyclic fatigue failure of the PAAm-alginate hydrogel, the samples of about 1 mm thick were tested using a DMA Q800 machine (TA Instruments). Samples were cyclically loaded in tension between zero and various stresses at a frequency of 1 Hz. Clamping devices on the machine were enclosed by a chamber equipped with a humidifier to prevent the hydrogel dehydration. Furthermore, samples were sprayed with water at regular intervals to ensure the fully swollen state. We applied different levels of cyclic stress in the range of 50–950 kPa. Maximum strain in the sample was continuously increasing up to final failure (Supplementary Fig. 6). The number of cycles to failure was recorded for each applied stress and the experimental data were plotted in the number of cycles to failure versus the applied stress (Fig. 3d). A line was fitted to the experimental data in the log-log scale which can be used to extrapolate the fatigue results to other stress levels.

### Measurement of optical and sonic properties

Refractive indices of water and the fully swollen PAAm-alginate hydrogel and Sylgard 184 were measured with a digital refractometer (Sper Scientific). Note that the refractive indices of Ecoflex and Elastosil were not able to be measured as both materials are optically opaque. Optical transmission spectra of water, the fully swollen PAAm-alginate hydrogel, Ecoflex, Elastosil and Sylgard 184 were measured using a spectrophotometer (BioMate; Thermo Scientific) with quartz cuvettes (10 mm optical travel length) for the whole range of visible light (400∼750 nm). A quartz cuvette with water was used as a reference to reduce the reflection from index mismatch. The transmittance is defined as *I/I*_0_, where *I* is the intensity of the transmitted light, and *I*_0_ is the intensity of the incident light (Fig. 4a).

In order to measure the speed of sound of the hydrogels and elastomers, the transmission ultrasound signals at three different frequencies (that is, 40 kHz, 200 kHz and 1 MHz) were used. All measurements were conducted in the water tank using the samples with size of 120 mm in width, 120 mm in length and 50 mm in thickness (Supplementary Fig. 7b). A pulse of central frequency (that is, 40 kHz, 200 kHz and 1 MHz) was generated by a signal generator (Tektronix) and sent to the transducer (CTG Model ITC-1042 for 40 kHz, Olympus NDT ultrasonic transducers for 200 kHz and 1 MHz). The signal passed through the sample and the transmitted signal was collected by the hydrophone (CTG Model ITC-1089D for 40 kHz, Olympus NDT ultrasonic transducers for 200 kHz and 1 MHz). The measured signals were sent to the computer through the connected oscilloscope (Agilent) and the data were processed by MATLAB. The data were plotted in the measured signal amplitude versus the travel time curves (Fig. 5a–c). The *t*=0 corresponded to the time at which the ultrasound signal was sent from the transducer and the signal amplitudes were measured by the hydrophone upon the arrival of the transmitted ultrasound signals through the samples. The ultrasound signals sent at each frequency had the same amplitude while the attenuation varied among sample materials due to different acoustic impedance and viscous effect of each material. At each frequency, the speed of sound in pure water was measured as a control parameter. The arrival time difference, *δ*_*t*_ of the received pulse after passing through the sample and through pure water was obtained (Fig. 5a–c) and the speed of sound in the sample was calculated as


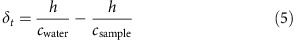


where *h* represents the thickness of the sample, *c*_*water*_ represents the speed of sound in pure water and *c*_sample_ represents the speed of sound in the sample.

The acoustic impedance, *z*_0_ of the hydrogels and elastomers were calculated from the definition of the acoustic impedance as


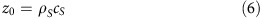


where *ρ*_*S*_ represents the known density of materials, and *c*_*S*_ represents the measured speed of sound of materials^36^. The acoustical reflection coefficient, *R* at the interface between water and the sample material can be calculated from the acoustic impedances of materials by


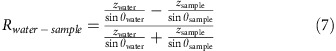


where *z*_water_ is the acoustic impedance of water, *z*_sample_ is the acoustic impedance of the sample material and *θ*_water_ and *θ*_sample_ are the incident angle of the plane sound wave with respect to the water and sample interfaces respectively. Note that the incident angle of the sound wave was perpendicular to the water and sample interfaces (that is, *θ*_water_=*θ*_sample_=90°) in the measurements (Supplementary Fig. 7b). The degree of acoustical refection of the projected sound wave at the interface between water and the sample material is determined by the acoustical reflection coefficient (Supplementary Fig. 7a)^29^,^36^. Ultrasound images of the hydrogel and elastomer actuators were taken by a commercial imaging probe (GE LOGIQ E9; GE Healthcare) using 4 MHz ultrasound pulse mode. To take ultrasound images of the sample in water, the samples were immersed into the large water tank filled with deionized water and the probe was placed in the tank above the samples. The contrast and other settings for the ultrasound image were first tuned for the elastomer actuators to clearly visualize the boundary of the sample, then the ultrasound images of the hydrogel actuators were taken without further changing the settings (Fig. 5d,e). Note that all optical and sonic measurements were performed under the room temperature condition (23 °C).

### Preparation of demos for hydraulic hydrogel actuators and robots

To demonstrate the swimming of the hydrogel robotic fish, the hydrogel robotic fish with two hydraulic actuators on each side was prepared (Supplementary Fig. 2b). The hydrogel robotic fish was connected to two separate sets of programmable syringes pumps to actuate both sides synchronously in water. A floating block (polystyrene foam) was used to stabilize the position of the actuator and allow it to swim (Supplementary Fig. 8a). The supply flow rate of 300 ml min^−1^ was used for both sides of hydraulic hydrogel actuators to generate fast tail stroke (that is, actuation frequency of 1 Hz). Very flexible soft silicone tubings were adopted as hydraulic connections and fixed to the water tank to minimize possible effects from hydraulic connections during swimming.

To demonstrate the ball-kicking by the hydrogel actuator, a rubber-ball (7.5 cm diameter) and the bending hydrogel actuator were used (Supplementary Fig. 2a). The full-bending hydrogel actuator was clamped to the fixture in a vertical position in water, then the rubber-ball was placed around the free end of the hydrogel actuator (Supplementary Fig. 8b).

To demonstrate the fish catching by the hydrogel gripper, the hydrogel gripper with six bending actuators was prepared (Supplementary Fig. 2c). The hydrogel gripper was actuated by a single hydraulic input connected to the syringe pumps. A live goldfish was placed in the water tank, and it was caught, lifted and released by the hydrogel gripper without harming it (Supplementary Fig. 8c). Note that all procedures conformed to the Guide for the Care and Use of Laboratory Animals and were approved by the Massachusetts Institute of Technology Institutional Committee on Animal Care (CAC).

### Data availability

The data that support the findings of this study are available from the corresponding author on request.

## Additional information

**How to cite this article:** Yuk, H. *et al*. Hydraulic hydrogel actuators and robots optically and sonically camouflaged in water. *Nat. Commun.* **8,** 14230 doi: 10.1038/ncomms14230 (2017).

**Publisher's note:** Springer Nature remains neutral with regard to jurisdictional claims in published maps and institutional affiliations.

## Supplementary Material

Supplementary Information Supplementary Figures

Supplementary Movie 1 Swimming of transparent leptocephalus in oceanic environment. The movie clip is credited to Barry Haythorne and Rob Rutgers from HRF U/W Production

Supplementary Movie 2 Actuation of the unit-segment hydraulic hydrogel actuator in water. The transparent hydrogel is dyed for better visual representation.

Supplementary Movie 3 Actuation of the bending hydraulic hydrogel actuator in water. The transparent hydrogel is dyed for better visual representation

Supplementary Movie 4 Actuation of the bending hydraulic hydrogel actuator in finite element simulation

Supplementary Movie 5 Swelling-driven osmotic actuation of a bulk hydrogel strip in water. A Sylgard 184 layer is attached on the hydrogel strip as an inextensible element. The hydrogel and Sylgard 184 are dyed for better visual representation

Supplementary Movie 6 Forward fish-like swimming of a hydrogel robotic fish in water. The hydrogel fish can keep the camouflaged state when swimming over rainbow-colored background owning to its optical transparency

Supplementary Movie 7 A transparent hydrogel actuator kicks a rubber-ball in water. The high-speed and high-force hydraulic actuation enables effective ball-kicking motion. Backgrounds of different colors are used to demonstrate the optical transparency of the hydrogel actuator.

Supplementary Movie 8 A transparent hydrogel gripper catches, lifts and releases a live goldfish. The agile actuation and optical transparency of the hydrogel gripper allow its successful catching of the goldfish. The gripper holds and then releases the caught goldfish without harm owning to the gripper's softness. Note that all procedures conformed to the Guide for the Care and Use of Laboratory Animals and were approved by the Massachusetts Institute of Technology Institutional Committee on Animal Care (CAC).

## Figures and Tables

**Table 1:** Comparison of optical and sonic properties of water, hydrogel and elastomers.

	**Water**	**Hydrogel**	**Ecoflex**	**Elastosil**	**Sylgard 184**
*n*Refractive index	1.3330	1.3365	NA*	NA*	1.4225
*I/I*_0_ Transmittance (relative to water)	100%	>90%	*<* 5%	<0.1%	>90%
*c* Speed of sound (m s^−1^)	1447.5	1485.7	983.4	979.6	1022.4
*z*_*0*_Acoustic impedance (Pa s m^−1^)	1.448 × 10^6^	1.487 × 10^6^	1.052 × 10^6^	1.058 × 10^6^	1.053 × 10^6^
*R*Acoustical reflection coefficient	0	0.013	0.158	0.156	0.158

^*^Ecoflex and Elastosil are optically translucent to opaque elastomers; therefore, their refractive indices are not available.
