# Supplementary material for: Hydraulic hydrogel actuators and robots optically and sonically camouflaged in water
Source: Nat Commun. 2017 Feb 1;8:14230. doi: 10.1038/ncomms14230 (PMC5296644; doi:10.1038/ncomms14230)
Supplement: Supplementary Information — Supplementary Figures [file ncomms14230-s1.pdf]

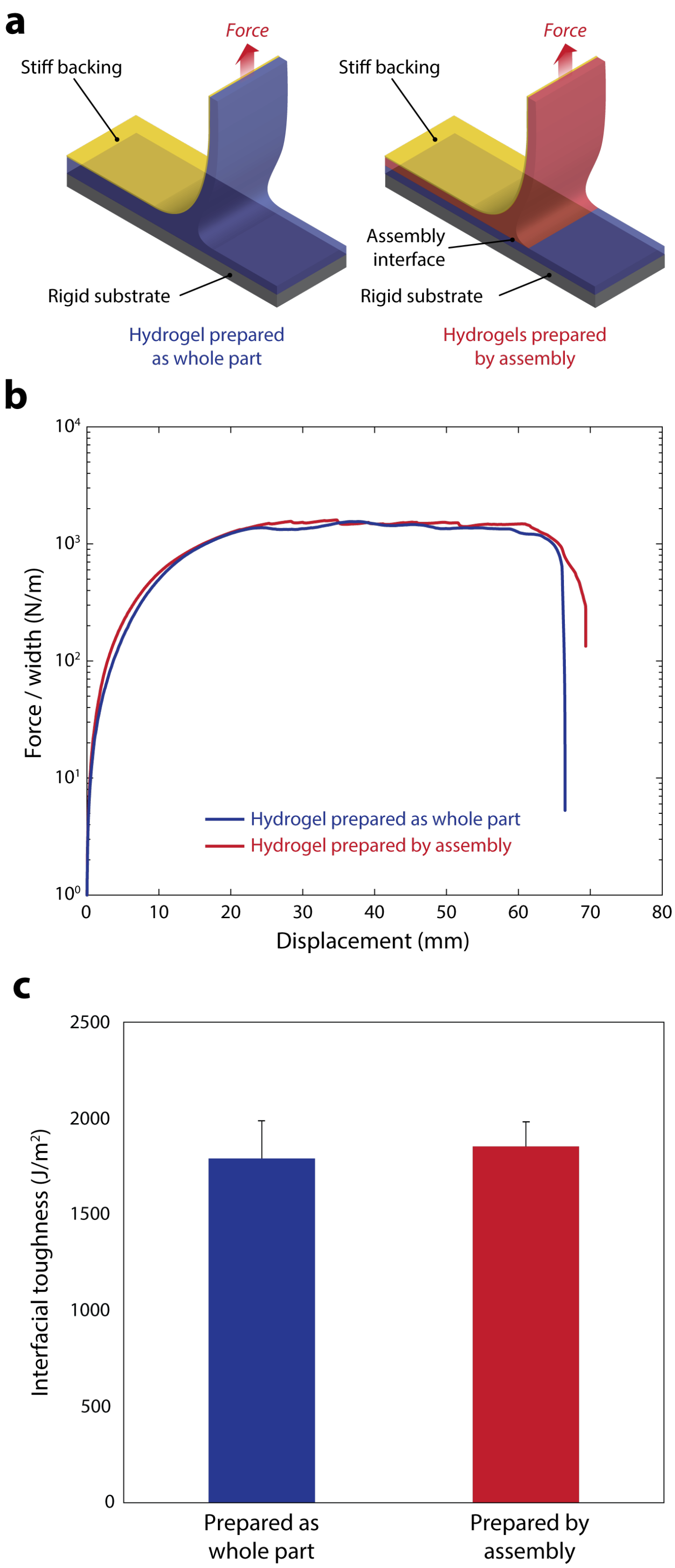
a
Force
Stiff backing
Rigid substrate
Hydrogel prepared
as whole part
Force
Stiff backing
Assembly
interface
Rigid substrate
Hydrogels prepared
by assembly
b
Force / width (N/m)
Displacement (mm)
Hydrogel prepared as whole part
Hydrogel prepared by assembly
c
Interfacial toughness (J/m²)
Prepared as
whole part
Prepared by
assembly

Supplementary Figure 1 | Robust interface between the assembled hydrogel parts. (**a**) Interfacial toughness is measured for PAAm-alginate tough hydrogel prepared as a whole part and PAAm-alginate tough hydrogel prepared by assembling shaped hydrogel parts. The interfacial toughness for both types of samples is measured by the standard 90-degree peeling test (ASTM D 2861). (**b**) Typical curves for the measured peeling forces per width of the hydrogel sheet for both types of samples. (**c**) The measured interfacial toughness for both types of samples. Error bars indicate 1 SD from three measurements.

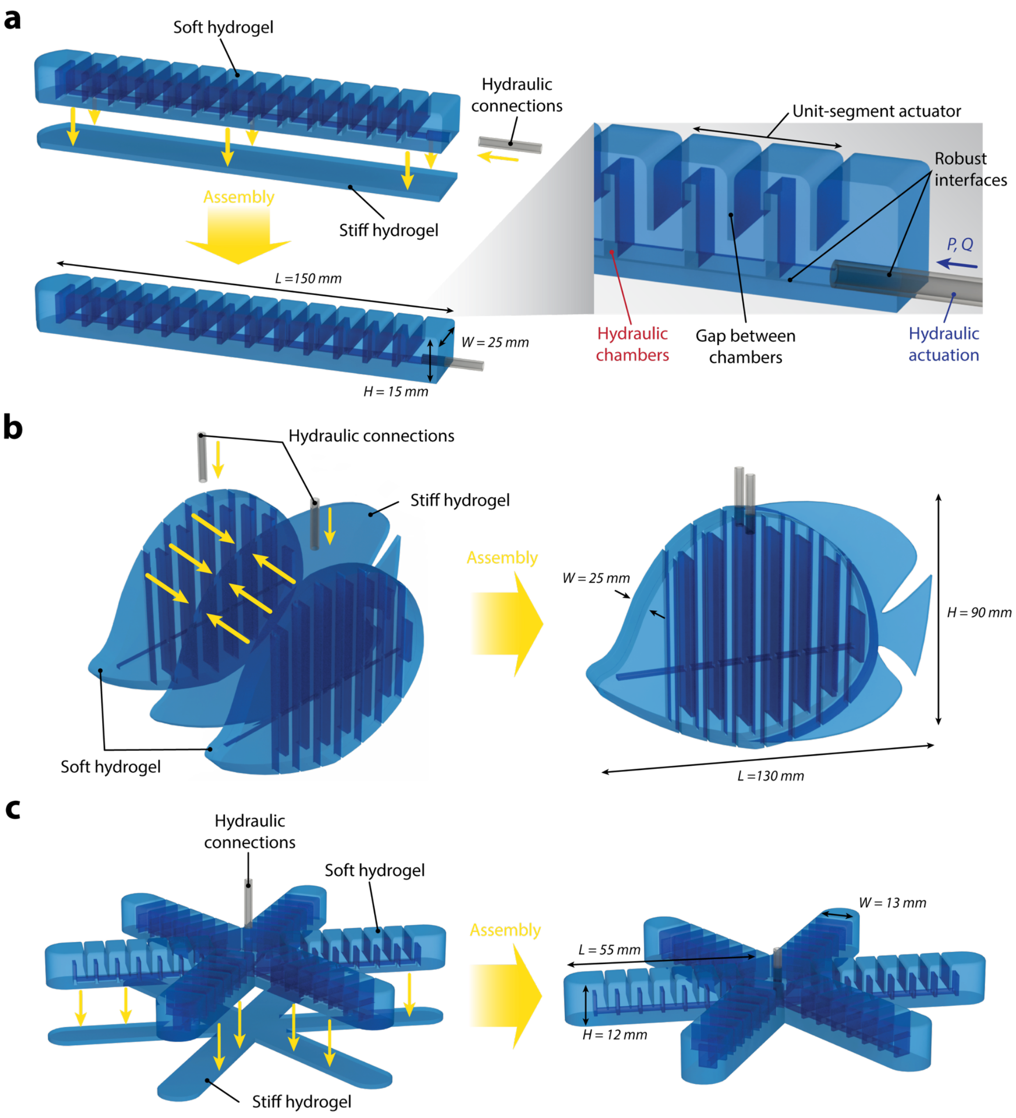

Supplementary Figure 2 | Design and dimensions of various hydrogel soft actuators and robots. (**a**) Design and dimensions of the bending actuator. The bending actuator consists of seven serially-connected unit-segment actuators. Soft hydrogel parts consist of hydraulic chambers and connections while the stiff hydrogel provides a less-extensible element to enable the bending under hydraulic actuation. (**b**) Design and dimensions of the hydrogel robotic fish. Hydraulic actuations on two sides of the fish are controlled by two separate hydraulic supplies. (**c**) Design and dimensions of the hydrogel gripper. Six bending actuators are actuated simultaneously by one hydraulic input.

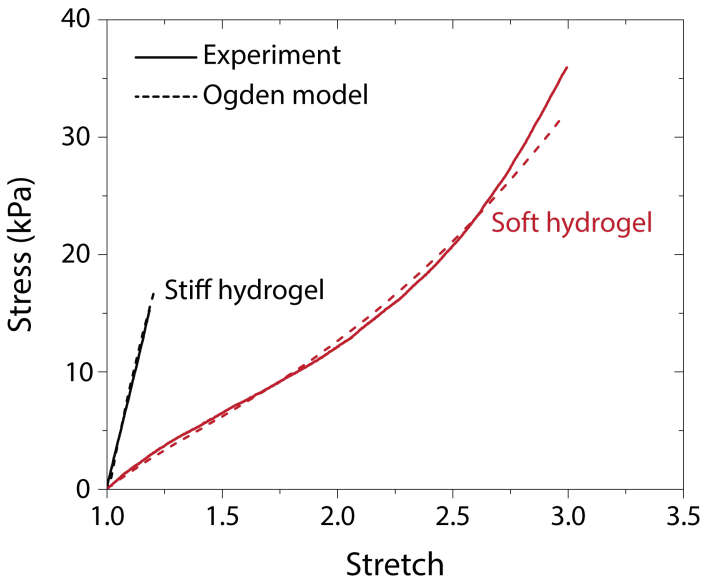

Supplementary Figure 3 | Nominal stress vs. stretch curves of stiff and soft hydrogels from experiments and fitted models. Both stiff and soft PAAm-alginate hydrogels have been deformed a few cycles up to the maximum deformation that will be achieved in the actuators and robots in this study. (i.e., $\lambda \sim 1.2$ for the stiff tough hydrogel and $\lambda \sim 2.7$ for the soft tough hydrogel).

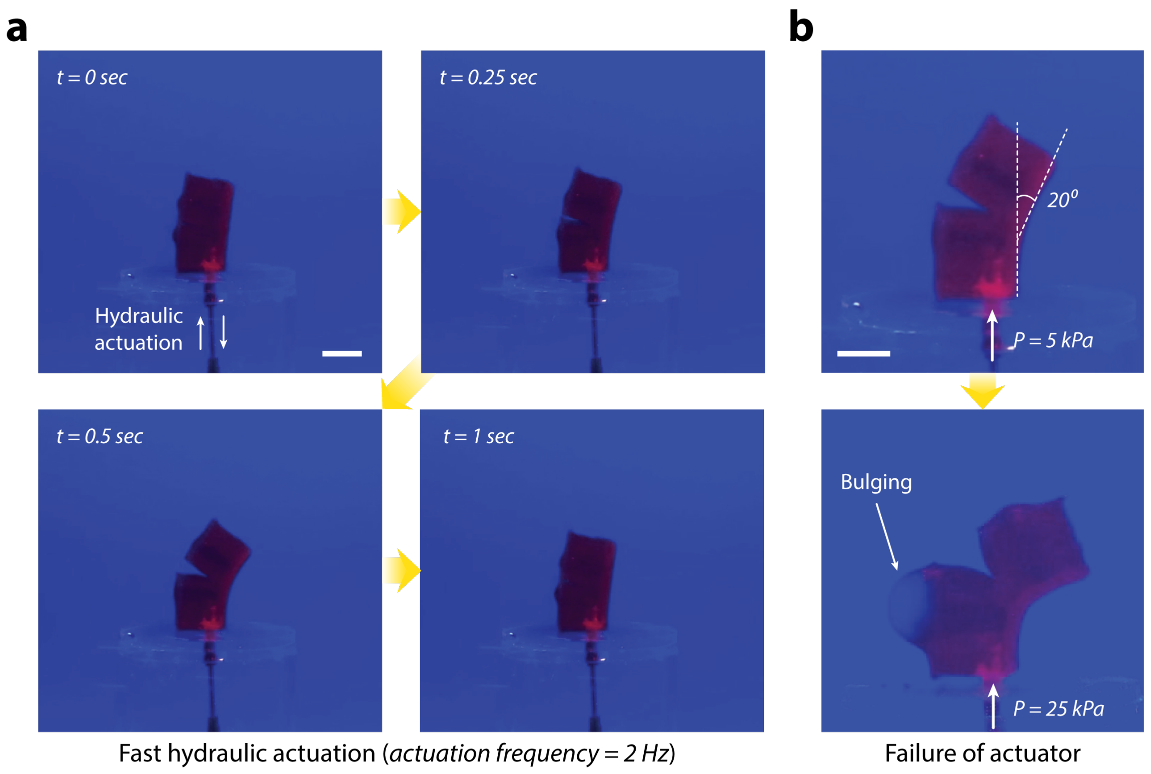

Supplementary Figure 4 | Fast hydraulic actuation and failure of the unit-segment actuator. (**a**) Fast bending actuation of the hydraulic hydrogel actuator with actuation frequency around 2 Hz. (**b**) The unit-segment hydrogel actuator undergoes failure under excessively high applied pressure by unstable inflation (e.g., bulging). Note that hydrogel actuators in **a**,**b** are dyed with color for better visual representation. Scale bars, 1 cm (**a**,**b**).

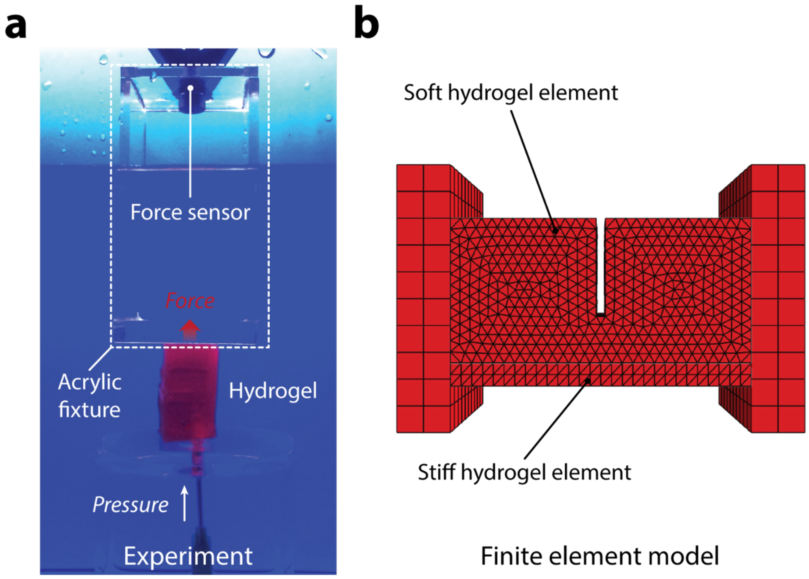

Supplementary Figure 5 | Experimental setup and finite element simulation for the force measurement on the unit-segment actuator. (**a**) Experimental setup for the force measurement. The force exerted by the unit-segment hydrogel actuator under increasing applied pressure is measured by a force sensor. (**b**) The same setup is modeled in finite element simulation. Note that hydrogel actuator in **a** is dyed with color for better visual representation.

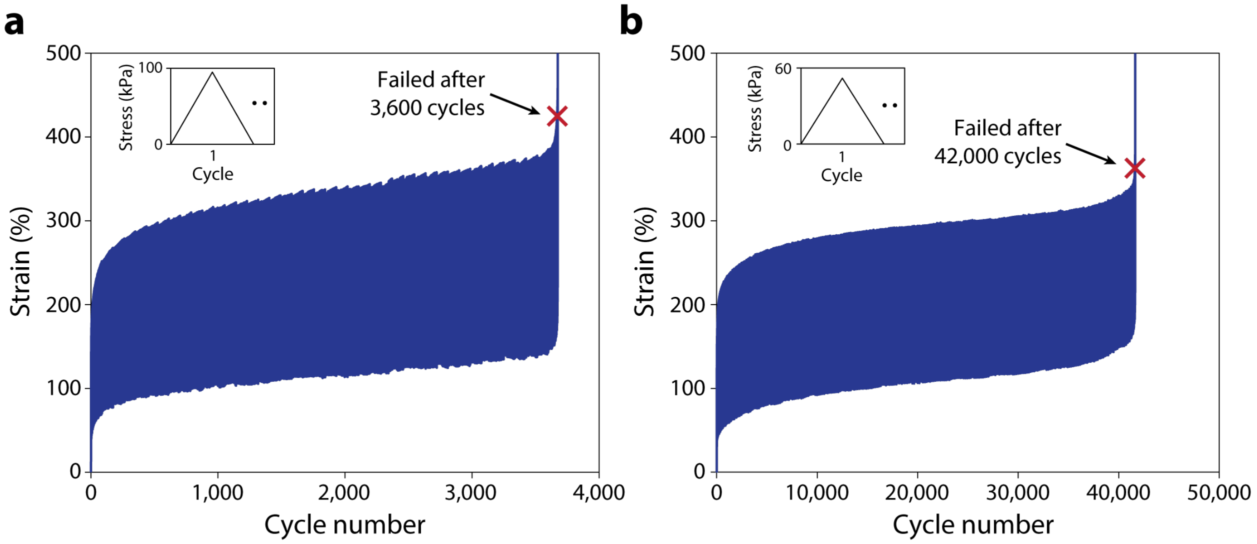

Supplementary Figure 6 | Strain evolution with cycles in stress-controlled cyclic fatigue tests of the hydrogel. (**a**) Cyclic nominal stress of 95 kPa leads to fatigue failure of the hydrogel after 3,600 cycles. (**b**) Cyclic nominal stress of 52 kPa leads to fatigue failure of the hydrogel after 42,000 cycles.

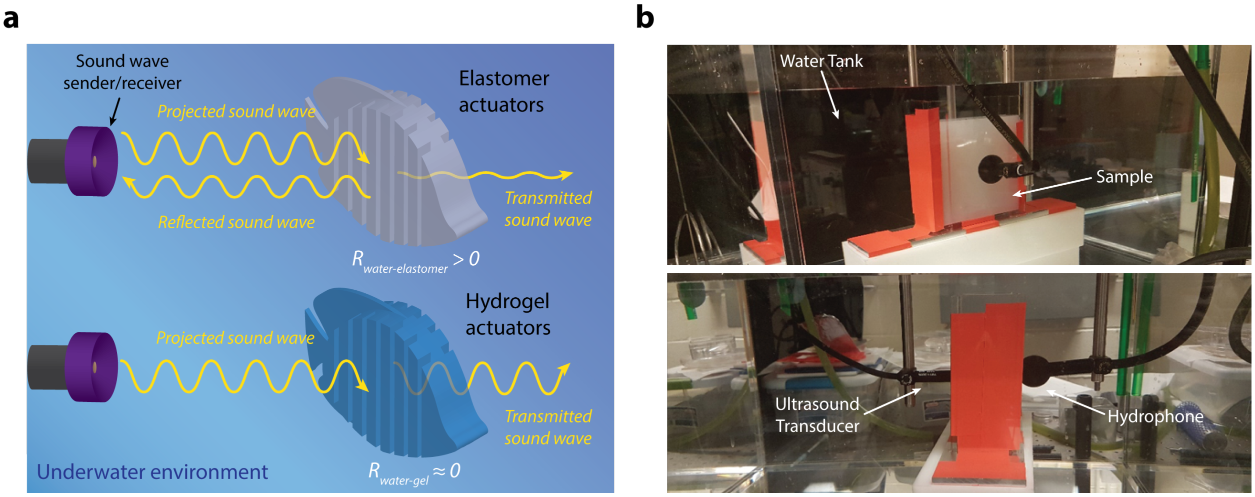

Supplementary Figure 7 | Sonic property measurements. (**a**) Schematic illustration of the difference in sonic transparency in elastomer and hydrogel actuators under water. (**b**) Speed of sound measurement setup for various materials. Samples are loaded within the water tank and ultrasound transducer and hydrophone are located at each side of the fixed sample.

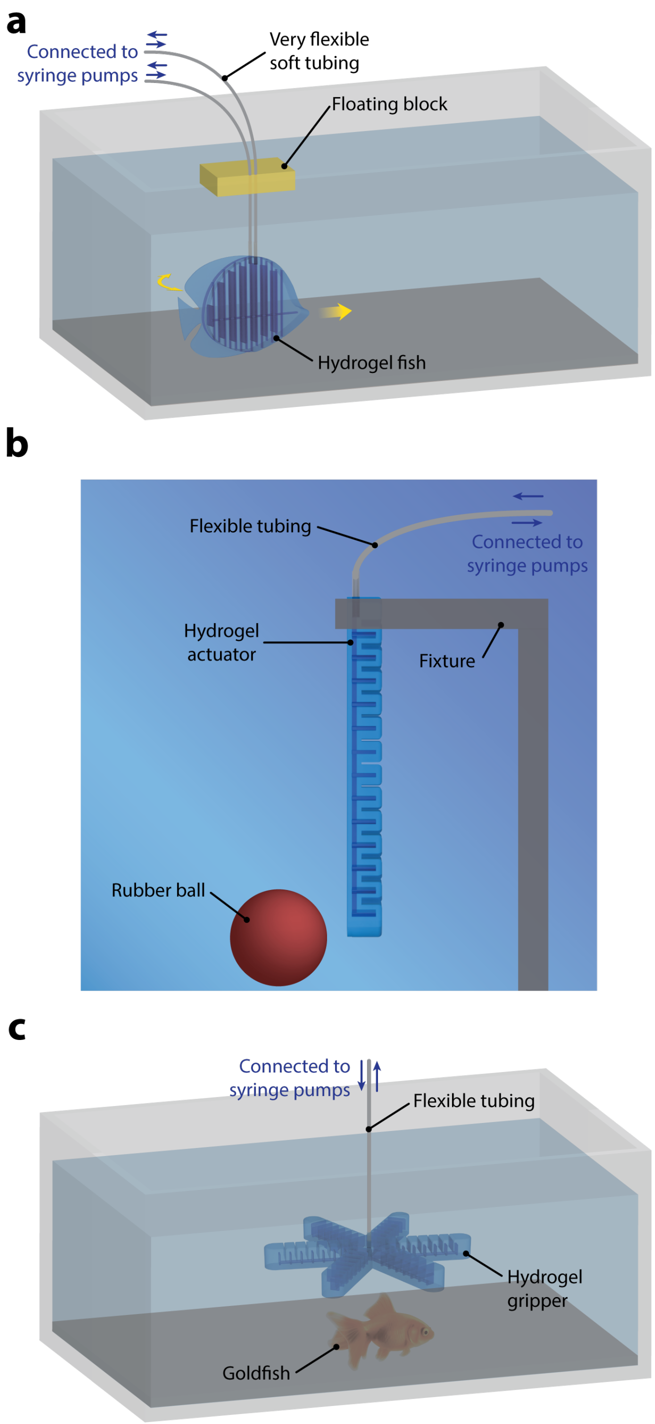

Supplementary Figure 8 | Experimental setups for demonstrations of hydrogel actuators and robots. (**a**) Experimental setup for swimming of the hydrogel robotic fish. Two sides of the

hydrogel fish are actuated by two separate hydraulic inputs connected to two sets of pumps. To endow positional stability, a floating block is connected to the flexible soft elastomeric tubings. (**b**) Experimental setup for kicking a rubber-ball by the bending hydrogel actuator. (**c**) Experimental setup for catching a live goldfish by the hydrogel gripper.
